# Three-dimensional visualization of arsenic stimulated mouse liver sinusoidal by FIB-SEM approach

**DOI:** 10.1007/s13238-016-0246-9

**Published:** 2016-02-08

**Authors:** Wenbo Li, Wei Ding, Gang Ji, Li Wang, Jianguo Zhang, Fei Sun

**Affiliations:** National Laboratory of Biomacromolecules, Institute of Biophysics, Chinese Academy of Sciences, Beijing, 100101 China; Center for Biological Imaging, Institute of Biophysics, Chinese Academy of Sciences, Beijing, 100101 China; College of Life Science, University of Chinese Academy of Sciences, Beijing, 100049 China

**Dear Editor,**

Liver sinusoidal endothelial cells (LSECs) are the most abundant non-parenchymal and highly specialized fenestrated cells in the liver. LSECs differ from endothelial cells of other capillaries due to the presence of open fenestrations and the absence of a basal lamina (Wisse et al., 1996; Braet and Wisse, 2002; Aird, 2007; Cogger et al., 2008). During the differentiation process, LSECs become fenestrated to facilitate the transfer of circulating nutrients, lipids, and lipoproteins between blood and the space of Disse for normal liver metabolism. Only particles smaller than the fenestrae can reach the hepatocytes or leave the space of Disse (Wisse et al., 1985). The size of fenestration changes upon different conditions to regulate the substrate exchange.

Previous studies reported that the diameter of the sinusoidal fenestrations varies between different species from 107 ± 1.5 nm (mean ± SEM) in the human liver (Wisse et al., 2008) to 141 ± 5.4 nm (mean ± SEM) in the mouse liver (Wisse et al., 2008), which was determined based on transmission electron micrographs of sections (Jacobs et al., 2010). However, considering the small size of fenestration, 100~150 nm in diameter, which is beyond the limit of resolution in light microscopy (Cogger and Couteur, 2009), in early stage, the morphology of fenestrations could only be studied primarily using conventional electron microscopy (Owen et al., 2010; Svistounov et al., 2012), however, with lack of 3D information.

Arsenic is a toxic metalloid and a common contaminant of drinking water. Drinking arsenic-contaminated water increases the risk of cardiovascular disease, lung disease, hepatic disease, and cancer in millions of people worldwide. Even at low levels, arsenic promotes angiogenesis and vascular remodeling in mice (Guha Mazumder, 2003; Mazumder, 2005; Navas-Acien et al., 2005). Pathological vascular remodeling, such as neovascularization, angiogenesis, and morphologic changes in vascular architecture, is a critical process in the development of vascular diseases including atherosclerosis, cardiovascular ischemic diseases, tumor vasculogenesis, and liver fibrosis. Different from angiogenesis of systemic vessels endothelial cells, LSECs angiogenesis, also called capillarization, is a dedifferentiation and maturation process with diagnostic hallmarks of LSEC defenestration and renewed surface expression of PECAM-1 and laminin-1 in response to environmental stress and aging (Straub et al., 2007). Liver capillarization precedes vascular remodeling of other liver vessels, such as hepatic arterioles and PBVP (peribiliary vascular plexus), causing blood flow shunting, vascular channel formation, and eventually liver fibrosis (Straub et al., 2007). Liver capillarization also affects the systemic vasculature and promotes atherogenesis by decreasing liver metabolism of lipids, lipoproteins, and glucose (Straub et al., 2007).

In this study, we investigated how the fenestration morphology of LSECs in mice changes in response to an arsenic stimulation. We utilized focused ion beam scanning electron microscopy (FIB-SEM) technique to reconstruct the 3D morphology (~20 × 20 × 1.5 μm^3^) of mouse liver sinusoidal with and without the arsenic stimulation. The three-dimensional data generated from FIB-SEM approach enabled us to perform morphologic comparison in 3D space and quantitative measurements and comparisons for the size and number of LSEC fenestrations.

FIB-SEM is a technique to generate high resolution three-dimensional images of biological samples in micrometer scale (Kizilyaprak et al., 2014). Samples are prepared by a similar method to transmission electron microscopy, typically by fixing the sample with aldehyde, staining with heavy metals such as osmium and uranium then embedding in an epoxy resin. The surface of the block of resin-embedded sample is imaged by detection of back-scattered electrons. Following imaging, the focused ion beam is used to trim a thin section (typically less than 30 nm) from the face of the block. After the section is trimmed, the sample block is raised back to the focal plane and imaged again. This sequence of sample imaging, section trimming and block raising can acquire many thousands of images with perfect alignment in an automated fashion and yield a 3D volume data of specimen.

The livers from both normal and As(III)-exposed mice (see Supplemental Materials and Methods) were firstly fixed, embedded in resin and sectioned for conventional transmission electron microscopy (TEM) examination. The region of the sinusoidal endothelium was selected and imaged. In consistency with the previous observation (Straub et al., 2007), the LSECs in normal mice contain numerous sieve plates with open fenestrae (Fig. 1A). While, the liver sinusoids in the As(III)-exposed mice become defenestrated with continuous endothelium membrane (Fig. 1B). Besides, the density of hepatocyte microvilli in the space of Disse, which protrude through the LSEC fenestrae, becomes significantly increased in the As(III)-exposed liver sinusoids (Fig. 1B).

**Figure 1:**
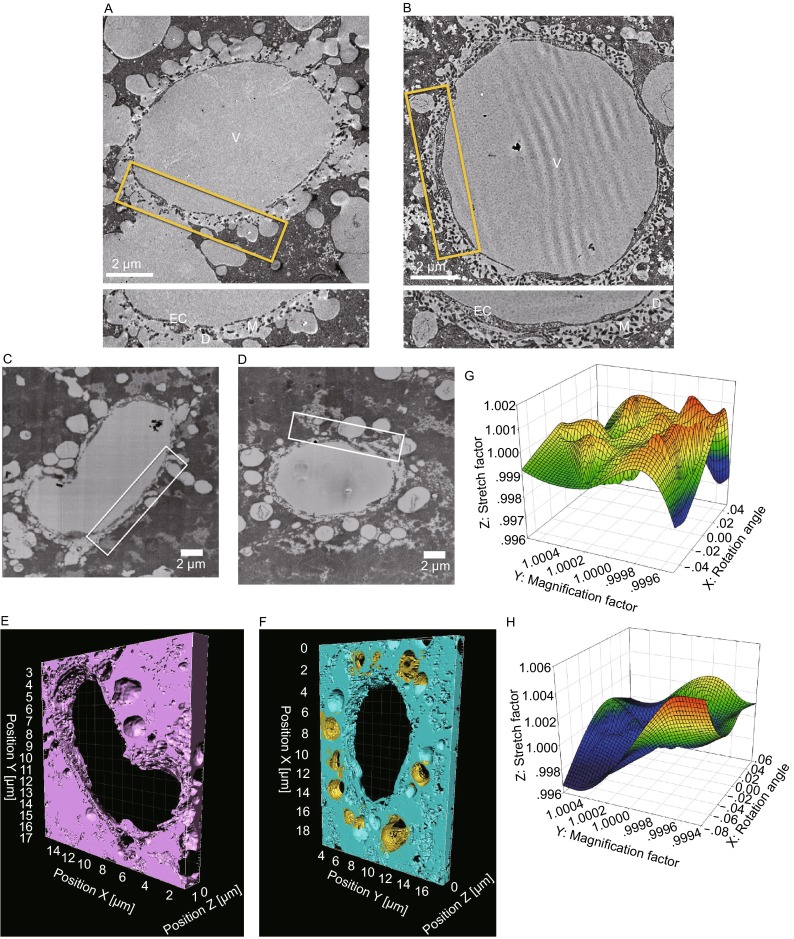
**Visualization of mouse liver sinusoidal by TEM and 3D reconstruction of mouse liver sinusoid by FIB-SEM**. (A) TEM micrograph of sinusoid in normal mice, which contain numerous sieve plates with open fenestrae. (B) TEM micrograph of sinusoid in arsenic treated mice, which show defenestration with continuous endothelium. The boxed region is zoomed in at the bottom. EC, endothelial cell; D, Disse; M, microvilli; V, sinusoidal vessel. (C and D) SEM micrographs of sinusoid in normal mice and arsenic treated mice respectively. (E and F) FIB-SEM reconstructions of sinusoid in normal mice and arsenic treated mice respectively. The scales in X, Y, and Z directions are labeled with the pixel size of 5 nm for XY direction and 15 nm for Z direction. (G and H) The statistic diagrams of the parameters including the image rotation angle (X axis), the image magnification correction factor (Y axis) and the image stretch correction factor (Z axis) during image alignment and deformation correction of serial SEM images of sinusoid in normal mice (G) and arsenic treated mice (H) respectively. These parameters are calculated from two sequential adjacent images (see also Fig. S1)

Since the size of fenestrations (>100 nm) is larger than the thickness of normal TEM sections (~70 nm), the overall morphology of fenestrations and their distribution along the sinusoidal vessel could not be resolved from a single TEM image. To get a more accurate observation of mouse liver sinusoidal fenestrations, we utilized focused ion beam scanning electron microscopy (FIB-SEM) technique to reconstruct the 3D morphology of mouse liver sinusoid in micrometer scale.

The SEM images of both normal and As(III)-exposed mouse liver sinusoid show perfect contrast and similar morphology observation (Fig. 1C and 1D) to that of TEM images (Fig. 1A and 1B). We collected 116 sequential FIB-SEM images (4096 × 4096) with the pixel size of 5 nm and the milling thickness of 15 nm to cover ~20 × 20 × 1.5 μm^3^ size of specimen for both normal and As(III)-exposed mouse liver sinusoid (Movies S1 and S2), respectively. After image alignment, denoising and reconstruction (see Supplemental Materials and Methods), the 3D morphology of mouse liver sinusoid was obtained (Fig. 1E and 1F), showing the morphology of LSECs and their fenestrations clearly in 3D space. Besides, the hepatocyte microvilli in the space of Disse are also well resolved. The small deformation factors for the rotation, magnification, and stretch corrections (Figs. 2G, 2H, and S1), which are less than 6%, 1%, and 1%, respectively, demonstrate the data collection stability of the FIB-SEM method and confirm the high quality of the 3D reconstructions.

**Figure 2:**
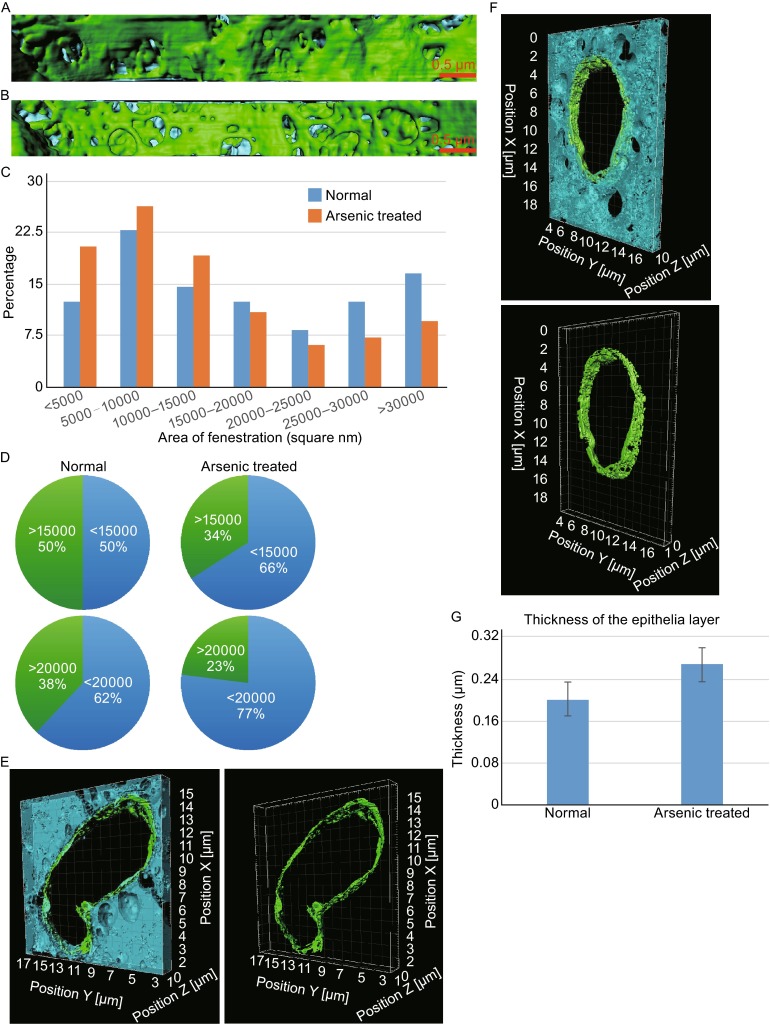
**3D** **Morphology comparison and quantitative analysis of fenestrations and epithelial cells layers between normal and arsenic treated specimen**. (A and B) 3D morphology of fenestrations in normal mice and arsenic treated mice respectively. The endothelial cells are colored in green and the hepatocyte microvilli are colored in cyan. (C) The histogram statistics of the numbers of fenestrations vs. their areas in normal mice (blue) and arsenic treated mice (orange) respectively. The numbers of fenestrations are normalized and shown in their percentages. (D) The statistics of the fenestration numbers larger or smaller than 15,000 nm^2^ (up) or 20,000 nm^2^ (bottom) for normal specimen (left) and arsenic treated specimen (right). (E and F) 3D morphology of sinusoid in normal mice (E) and arsenic treated mice (F) with the epithelia layer colored in green and segmented in right. The scales in X, Y, and Z directions are labeled with the pixel size of 5 nm for XY direction and 15 nm for Z direction. (G) Comparison of the averaged thickness of the epithelial layers for the normal mice and arsenic treated mice. See Supplemental Materials and Methods for the details of epithelial layer thickness measurements, sampling, and averaging

At the current experiments, the pixel size of each FIB-SEM image is about 5 nm in XY direction and the FIB cutting step size is 15 nm in Z direction, and the size of live sinusoid fenestrations are approximately 50–150 nm in diameter, thus the fenestrations can be well resolved after the reconstructions by FIB-SEM method (Fig. 2A and 2B). In normal mice, most fenestrations are irregular holes with a relative large size (Fig. 2A) while, in As(III)-exposed mice, a majority of fenestrations appear as approximately round holes and most of them are relatively smaller than those in normal ones (Fig. 2B).

Based on the 3D morphologies of the liver sinusoids (Fig. 1E and 1F), we were able to count the number of fenestrations and measure their sizes statistically. Since the fenestration is irregular, we utilized a minimal rectangle to cover each fenestration and used the area of that rectangle as the approximate determinant of the fenestration size. From the statistic histogram of the numbers of fenestrations vs. their sizes, we could observe the different distributions of the fenestrations for the normal and As(III)-exposed specimen. For the normal specimen, the size distribution of the fenestrations is relatively uniform, while, for the As(III)-exposed specimen, more fenestrations tend to have a smaller size (Fig. 2C). We further counted the percentages of fenestrations in different sizes. For the normal specimen, the percentage of fenestrations with the size smaller than 15,000 nm^2^ is 50% and 62% for the size smaller than 20,000 nm^2^ (Fig. 2D). However, for the As(III)-exposed specimen, the percentage of fenestrations with the size smaller than 15,000 nm^2^ is increased to 66% and 77% for the size smaller than 20,000 nm^2^ (Fig. 2D). In total, the average area of fenestrations is about 18,000 nm^2^ for the normal specimen and about 10,000 nm^2^ for the As(III)-exposed one. As the result, the above quantitative analysis of the 3D morphologies of the liver sinusoids clearly reveals that the liver sinusoids become defenestrated upon As(III)-exposure.

The thicknesses of the epithelia layers are visually different between normal and As(III)-exposed specimen according to both TEM (Fig. 1A and 1B) and FIB-SEM (Fig. 2E and 2F) analyzes. We further measured the averaged thicknesses of the epithelia layers quantitatively and got the measurements of 200 nm and 270 nm for the normal and As(III)-exposed specimen, respectively (Fig. 2G). As a result, these comparisons suggest that after As(III)-exposure the thickness of the epithelia layers of live sinusoids is increased.

FIB-SEM has been recently emerging as an efficient technique to study the 3D high-resolution ultrastructure of cells and tissues by acquiring thousands of sequential surface images automatically in a perfect alignment. Here, we utilized FIB-SEM technique to achieve 3D reconstruction of mouse liver SEC ultrastructure within 1.5 µm scale and 15 nm step size in Z direction and 5 nm pixel size in XY plane, and observed the ultra-structural changes upon the arsenic stimulation. By comparing the normal specimen with the As(III)-exposed one in three-dimensional space, we found that after As(III)-exposure, the sizes of live sinusoid fenestrations are reduced and the thicknesses of the epithelia layers are increased. Our present results provided a further support to understand the molecular mechanism of LSECs regulation and established a state-of-the-art approach to investigate LSECs in a large scale.

## Electronic supplementary material

Supplementary material 1 (DOCX 285 kb)

Supplementary material 2 (AVI 1594 kb)

Supplementary material 3 (AVI 1889 kb)
