# Supplementary material for: Three-dimensional visualization of arsenic stimulated mouse liver sinusoidal by FIB-SEM approach
Source: Protein Cell. 2016 Feb 8;7(3):227–32. doi: 10.1007/s13238-016-0246-9 (PMC4791422; doi:10.1007/s13238-016-0246-9)
Supplement: Supplementary file 1 — Supplementary material 1 (DOCX 285 kb) [file 13238_2016_246_MOESM1_ESM.docx]

**SUPPLEMENTAL MATERIALS AND METHODS**

**Animal facility**

Animal exposures were performed in agreement with institutional guidelines for animal safety and welfare at Institute of Biophysics, Chinese Academy of Sciences. C57BL/6N male mice, aged 6-8 weeks weighing approximately 20 g were obtained from Institute of Experimental Animals, Chinese Academy of Medical Sciences. Standard mouse chow and drinking water solutions were fed ad libitum for 5 weeks to mice. Fresh drinking water solutions containing 250 ppb sodium arsenite were prepared triweekly using commercially bottled drinking water according to (Straub et al., 2007).

**Mouse liver perfusion and chemical fixation for TEM**

Mouse livers were cleared by perfusion with PBS (1 mL/min) through the inferior venacava, perfusion fixed with 3 mL 2.5% glutaraldehyde (GA) in PBS, cut, and immersed in 2.5% GA for 2 days at 4°C. Samples were processed for transmission electron microscopy (TEM) as described previously (Ayache, 2010). Briefly, several 1-2 mm^3^ GA-fixed cubes were harvested, washed in PBS, and post-fixed in aqueous 1% OsO_4_,1% K_3_Fe(CN)_6_ for 1 hr. After several times of washes by PBS, pellets were dehydrated through a graded series of 30% to 100% ethanol, 100% propylene oxide, and infiltrated for 1 hr in a 1:1 mixture of propylene oxide: polybed 812 epoxy resin (Polysciences, Warrington, PA). After several changes of 100% resin over 24 hrs, pellets were embedded in molds, cured at 37°C overnight, and then hardened for 2 days at 65°C.

The block of resin-embedded specimen was trimmed and sectioned by ultra-microtome (UC6, Leica). Thick sections (300 nm) were stained with toluidine blue solution (0.5% toluidine blue, 1% sodium borate) for observation in light microscopy (Olympus Provis, Malvern, PA). Ultra-thin sections (70 nm) were prepared and stained with Uranyl acetate (UA) and lead citrate before observation in TEM.

**Mouse liver perfusion and chemical fixation for FIB-SEM**

Animals were anesthetized and perfused with normal Ringer’s solution containing xylocaine (0.2 mg/ml) and heparin (20 units/ml) for 2 minutes at 35°C followed by 0.15 M cacodylate buffer (Ted Pella Inc., Redding, CA) pH 7.4 containing 2.5% glutaraldehyde (Electron Microscopy Sciences, Hartfield, PA), 2% paraformaldehyde (Electron Microscopy Sciences, Hartfield, PA) with 2mM calcium chloride at 35°C for 5 minutes. Liver tissues were then removed and fixed for an additional 2-3 hrs on ice in the same solution.

The liver was cut into small (< 2 mm^3^ x 2 mm^3^) pieces with a razor blade. Tissues were washed 5 x 3 minutes in cold cacodylate buffer containing 2 mM calcium chloride. Right before use, a solution containing 3% potassium ferrocyanide and 4 mM calcium chloride in 0.3 M cacodylate buffer is combined with an equal volume of 4% aqueous osmium tetroxide (Electron Microscopy Sciences, Hartfield, PA). The tissues were incubated in this solution for 1 hr on ice.

At the same time, a fresh TCH (thiocarbohydrazide) solution was prepared. 0.1 g thiocarbohydrazide (Ted Pella) was added to 10 ml ddH_2_O and agitated by swirling gently every 10 minutes in a 60° C oven until complete dissolving. The solution was filtered through a 0.22 um Millipore syringe filter right before use.

After the first round of osmium tetroxide fixation, the tissues were washed with ddH2O at room temperature 5 x 3 minutes (~15 minutes total) and then placed in TCH solution for 20 minutes at room temperature. Tissues were then rinsed again 5 x 3 minutes in ddH_2_O at room temperature and thereafter placed in 2% osmium tetroxide in ddH_2_O for 30 minutes at room temperature. Then the tissues were washed 5 x 3 minutes at room temperature in ddH_2_O before placed in 1% uranyl acetate (aqueous) and left in a refrigerator (~4°) overnight.

In the next day, en bloc Walton’s lead aspartate staining was performed (Walton, 1979). The lead aspartate solution was made by dissolving 0.998 g of L-aspartic acid (Sigma-Aldrich) in 250 ml of ddH_2_O as a stock aspartic acid stock solution and then dissolving 0.066 g of lead nitrate in 10 ml of aspartic acid stock solution with pH adjusted to 5.5 using 1 M KOH. The lead aspartate solution was placed in a 60°C oven for 30 minutes for a complete dissolving. The tissues were washed 5 x 3 minutes in ddH_2_O at room temperature and then incubated with the lead aspartate solution in the 60°C oven for 5 minutes. The followed sample preparation (washing in ddH_2_O, dehydration, infiltration and embedding) was performed in the same way as above for TEM preparation.

**Data collection in FIB-SEM**

The block of resin-embedded specimen was placed in the chamber of Helios NanoLab 600i Dual beam SEM (FEI, Netherland). By using the gas injection system (GIS), an additional layer of Pt was deposited on the ROI (region of interest) to protect the specimen surface from ion beam damage. First, the stage was tilted 54° to allow the specimen surface to be perpendicular to the focused ion beam (FIB). To create a surface to be imaged in SEM, a trench was milled just near the Pt covered area and a high ion current (6.5–10 nA) was used to reduce the preparation time. Then, the FIB was set up to mill the Pt covered area with an ion current of 0.79 nA and the milling thickness of ~15 nm. After FIB milling, the SEM (operated at 3 kV) was used to image the ROI below the Pt layer with the electron probe size of 5 nm. The dwell time was adjusted to keep the overall imaging time less than 1 min. The FIB milling and SEM imaging were repeated again to get the image of another new milled block surface. There are 116 slice images collected for both normal and As(III)-exposed specimen, respectively.

**Image processing and analysis**

The FIB-SEM images were firstly contrast-enhanced by CLAHE (Zuiderveld, 1994), denoised by the median filter with the radius of 2 pixel in ImageJ (Schindelin et al., 2012), and then aligned by “ImageJ Linear Stack Alignment with SIFT” (Schindelin et al., 2012) and “IMOD Automatic alignment“ (Lowe, 2004). The rotation angle, magnification correction factor and stretch correction factor of each image by comparing its sequential adjacent image were calculated and saved for further analysis. The three-dimensional reconstruction and rendering were performed by using IMARIS (OLYMPUS, Japan). The statistical differences of the fenestration areas and epithelia layer thicknesses between the normal and As(III)-exposed specimen were analyzed according to the following rules.

Since the fenestration is not a regular circle, we firstly covered it by looking for a minimal rectangle and thereafter used the area of the rectangle as the approximate measurement of the fenestration size.

The thickness of the sinusoid epithelia layer is calculated by the formula.

$$T=\frac{V}{H*\bar{P}}$$

$$H=h*N$$

$$\bar{P}=(\sum_{i=1}^{N} p_{i})/N$$

Where, $T$: the mean thickness of the sinusoid epithelia layer; V: the volume of the inner wall that can be calculated by IMARIS automatically; H: the thickness of the reconstructed volume; h: the thickness of each slice; N: the number of slices; $\bar{P}$: the mean perimeter of the epithelia layer of the whole sinusoid lumen. $p_{i}$: The perimeter of the sinusoid epithelia layer in each slice.

**SUPPLEMENTAL FIGURES**

**
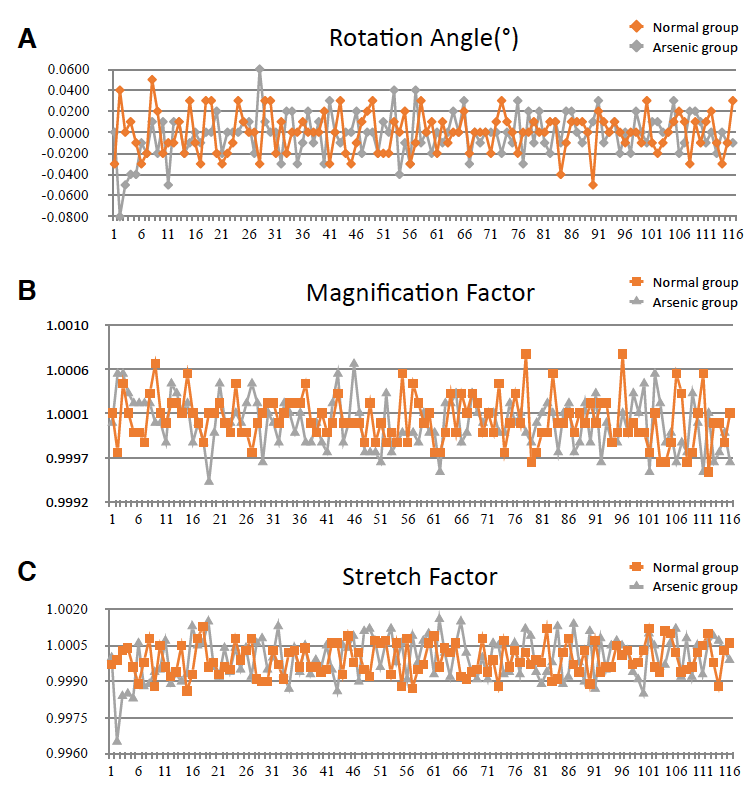
**

**Figure S1**. **The plots of image alignments and deformation corrections for serial SEM images of sinusoid in normal mice and arsenic treated mice**. (**A**) The plot for the image rotation angle. (**B**) The plot for the image magnification correction factor. (**C**) The plot for the image stretch correction factor. Each data point represents one SEM image. These parameters are calculated from two sequential adjacent images.

**SUPPLEMENTAL MOVIES LEGENDS**

**Movie S1**. A video of Z-slicing view of FIB-SEM reconstruction of liver sinusoid of normal specimen. The video contains 116 frames with each frame representing one sequential SEM image that has been well aligned and deformation-corrected. The Z-thickness of each SEM image is 15 nm. The contrast of each SEM image has been inverted from its raw form to keep the real structural information in black while the background in white.

**Movie S2**. A video of Z-slicing view of FIB-SEM reconstruction of liver sinusoid of arsenic treated specimen. The video contains 116 frames with each frame representing one sequential SEM image that has been well aligned and deformation-corrected. The Z-thickness of each SEM image is 15 nm. The contrast of each SEM image has been inverted from its raw form to keep the real structural information in black while the background in white.

**SUPPLEMENTAL REFERENCES**

Ayache, J. (2010). Sample preparation handbook for transmission electron microscopy : methodology (New York, Springer).

Lowe, D.G. (2004). Distinctive image features from scale-invariant keypoints. International journal of computer vision 60, 91-110.

Schindelin, J., Arganda-Carreras, I., Frise, E., Kaynig, V., Longair, M., Pietzsch, T., Preibisch, S., Rueden, C., Saalfeld, S., and Schmid, B. (2012). Fiji: an open-source platform for biological-image analysis. Nature methods 9, 676-682.

Straub, A.C., Stolz, D.B., Ross, M.A., Hernández‐Zavala, A., Soucy, N.V., Klei, L.R., and Barchowsky, A. (2007). Arsenic stimulates sinusoidal endothelial cell capillarization and vessel remodeling in mouse liver. Hepatology 45, 205-212.

Walton, J. (1979). Lead asparate, an en bloc contrast stain particularly useful for ultrastructural enzymology. J Histochem Cytochem 27, 1337-1342.

Zuiderveld, K. (1994). Contrast limited adaptive histogram equalization. Paper presented at: Graphics gems IV (Academic Press Professional, Inc.).
